# The British *E. coli* O157 in cattle study (BECS): factors associated with the occurrence of *E. coli* O157 from contemporaneous cross-sectional surveys

**DOI:** 10.1186/s12917-019-2188-y

**Published:** 2019-12-05

**Authors:** Madeleine K. Henry, Catherine M. McCann, Roger W. Humphry, Mair Morgan, Alice Willett, Judith Evans, George J. Gunn, Sue C. Tongue

**Affiliations:** 1 Epidemiology Research Unit (Inverness campus), Scotland’s Rural College (SRUC), Kings Buildings, West Mains Road, Edinburgh, EH9 3JG UK; 2 RSK ADAS Ltd., Spring Lodge, 172 Chester Road, Helsby, Cheshire, WA6 0AR UK

**Keywords:** Cattle, *Escherichia coli* O157, Risk factors, Super-shedder, STEC, Public health

## Abstract

**Background:**

*Escherichia coli* O157 is a bacterial pathogen associated with severe disease in humans for which cattle are an important reservoir of infection. The identification of possible risk factors for infection in cattle could facilitate the development of control strategies and interventions to mitigate the risk to human health. The purpose of this study was to utilize data collected in 2014–2015 during the two contemporaneous cross-sectional surveys of the British *E. coli* O157 in Cattle Study (BECS) to investigate potential risk factors for *E. coli* O157 status in cattle destined for the food chain.

**Results:**

In the England & Wales survey only one variable, herd size, was associated with the outcome farm-level *E. coli* O157 positive status. The odds increased for each additional animal in the herd. In the Scotland survey, as well as a measure of herd size (the number of cattle aged 12–30 months), having brought breeding females on to the farm in the last year also increased the odds, whereas farms sampled in spring were less likely to be positive compared to those sampled in autumn.

On the positive farms, in both surveys, an increase in the proportion of pats positive for *E. coli* O157 was associated with animals being housed at the time of sampling. However, the effect of housing on pat-level prevalence within positive groups was lower on farms from England & Wales than from Scotland (OR 0.45 (95% C.I. 0.24–0.86)).

**Conclusion:**

For the first time, factors associated with farm-level *E. coli* O157 status have been investigated in two contemporaneous surveys with comparable study design. Although factors associated with farm-level *E. coli* O157 status differed between the two surveys, one consistent factor was an association with a measure of herd size. Factors associated with the proportion of *E. coli* O157 positive pats within a positive farm were similar in both surveys but differed from those associated with farm-level status. These findings raise the hypothesis that measures to protect public health by reducing the risk from cattle may need to be tailored, rather than by assuming that a GB-wide protocol is the best approach.

## Background

Shiga toxin-producing *Escherichia coli* (STEC) – also called verocytotoxin-producing *E. coli* (VTEC) – such as *E. coli* O157 are well-known human pathogens causing significant morbidity in both developed and developing countries. STEC can cause a range of illness from mild diarrhoea to haemorrhagic colitis, thrombotic thrombocytopaenic purpura and haemolytic uraemic syndrome (HUS). Haemolytic uraemic syndrome, which may be fatal, is a leading cause of acute kidney failure in children in Western countries [1]. The main virulence characteristic of STEC strains is the production of Shiga toxins, of which there are two major types, stx1 and stx2. These are further categorised into additional subtypes [2, 3]. Cattle – asymptomatic carriers of *E. coli* O157 – and other ruminant animals and their environment are important reservoirs for infection [4, 5].

Variations in the level of faecal shedding among cattle have been observed. Most animals shed only transiently low levels (< 100 colony-forming units per gram (CFU g^− 1^) faeces) of *E. coli* O157, while a small number of cattle excrete large quantities of the pathogen or shed lower levels over longer periods of time [6]. The term super-shedder was first introduced by Matthews et al. [7] and was defined by Chase-Topping et al. as an animal that excretes ≥10^4^ CFU of *E. coli* O157 g^− 1^ faeces [8].

The aim of analyses to identify possible risk factors for the presence of pathogens, such as *E. coli* O157, is to understand more about their frequency and patterns of distribution in the specified population. Knowledge of their frequency and distribution helps to form hypotheses about both possible causal relationships and transmission dynamics. These hypotheses can then be investigated further with the ultimate aim to determine potential control or risk mitigation measures [9].

Although there have been a number of studies in beef and dairy cattle in which farm management risk factors associated with faecal shedding of *E. coli* O157 have been investigated, to date, no consistent associations have been found [10]. This may be due to different study designs over different time periods, in different populations, with different management systems. Factors found to be associated with shedding include: large numbers of finishing cattle [11]; the presence of pigs on farm [11–13]; farms classed as dairy units stocking beef animals [11]; keeping cattle in pens compared to those group-housed or at pasture [14] and cattle being housed at the time of sampling [15]. In a study of finishing cattle in Scotland, spreading slurry (versus manure) on grazing land was also found to be a risk factor for the presence of *E. coli* O157 on-farm [11].

Studies of the seasonality of shedding have also produced mixed results. In Denmark the risk of excreting *E. coli* O157 was found to be highest in summer (June and September) [16], whilst the winter period (December to February) and periods of high rainfall, leading to hide contamination with faeces, have been identified as risk factors in England and Wales [14] and Australia [17], respectively.

The presence within cattle groups of individuals shedding high levels of *E. coli* O157 has been shown to affect the probability that *E. coli* O157 will be isolated from other cattle within the same group. In a study of rectal carriage of *E. coli* O157 in groups of slaughtered cattle, in which high-level carriers were defined as animals with faecal *E. coli* O157 levels of ≥10^3^ CFU g^− 1^ [18], cattle were more likely to be found to be carrying *E. coli* O157 if there were other cattle in their group displaying high-level carriage of *E. coli* O157, compared to groups where no high-level carriers were identified. Similar findings were found in a study investigating risk factors for high-level or super-shedding on Scottish farms [19].

The British *E. coli* O157 in Cattle Study (BECS) [20] provides the first opportunity to investigate factors associated with the occurrence of *E. coli* O157 at farm-level and within sampled cattle groups across Great Britain, in contemporaneous surveys with similar designs. We investigated the association of a variety of management and demographic factors with three different outcomes: firstly the presence of *E. coli* O157 among cattle groups on farms in Scotland and in England & Wales (Outcome 1); secondly the proportion of faecal pats positive on *E. coli* O157 positive farms (Outcome 2); and thirdly the probability of at least one pat from *E. coli* O157 positive farms being classed as a super-shedder (Outcome 3). The aim was to investigate if there are risk factors that could potentially inform local management measures and national policy approaches to mitigate the risk of this important zoonosis.

## Results

The final data sets comprised questionnaire and laboratory data from 110 farms in Scotland and 159 farms in England & Wales. Although 160 farms in England & Wales had been visited as part of the original study [20], all questionnaire data were missing for one farm; therefore, the risk factor analysis used data for the 159 farms in England & Wales for which there were complete data. Laboratory results from 2763 pat samples from Scotland and 2866 pat samples from England & Wales were used in this analysis.

In Scotland, 26 (23.6%) of 110 farms had at least one *E. coli* O157 positive faecal pat – i.e. they were defined as positive farms – and nine (34.6%) of these positive farms had at least one super-shedder pat [20]. In England & Wales 34 (21.4%) of 159 farms had at least one *E. coli* O157 positive pat and seven (20.6%) of these positive farms had at least one super-shedder pat [20]. The mean proportion of pats testing positive for *E. coli* O157 on positive farms (Outcome 2 investigated in this study) was 0.46 (95% Confidence Interval (CI) 0.32–0.60) in Scotland and 0.32 (95% CI 0.21–0.42) in England & Wales.

### Potential risk factor screening

There were 26 potential risk factors (PRFs) (see Table 1 for details). Odds ratios (OR) and *p*-values for those individual variables that were found to have a statistically significant univariable association (*p* ≤ 0.20) with a given outcome of interest in at least one of the surveys are provided as additional tables within supplementary information (Additional files 1, 2 and 3). All significant tests of association between pairs of PRFs that were individually associated with Outcomes 1 to 3 are provided as an additional table in supplementary information (Additional file 4).

**Table 1:** List of Potential Risk Factors (PRFs) selected for screening process

PRF	Explanation	Comments
*total cattle*	Total number of cattle on the farm on sampling day	Continuous
*management type*	Cattle management type the respondent indicated as primary system on the farm	Categorical, 4 levels; Dairy (used as baseline level in the analysis); Other, Suckler Beef; Specialist Finisher.
*AHD*^a^	Animal Health Division – a non-current geographical designation in Scotland	Categorical, 6 levels
*cattle 12–30 months*	Total number of cattle on the farm aged between 12 and 30 months on sampling day	Continuous
*cattle less than 1 year*	Total number of cattle on the farm aged under 1 year on sampling day	Continuous
*group size*	Total number of cattle in the sample group	Continuous
*oldest in group*	Age (months) of the oldest sample group animal	Continuous
*youngest in group*	Age (months) of the youngest sample group animal	Continuous
*season*	Calendar year divided into three-month periods with March–May = spring, etc.	Categorical, 4 levels: Autumn (used as baseline level in the analysis); Winter, Spring; Summer
*housed*	Whether the sample group was completely housed or had access to grazing	Dichotomous; baseline “grazing”
*feed changed*	Whether or not the sample group’s feed had changed in the 2 weeks before sampling	Dichotomous; baseline “no”
*location changed*	Whether or not the sample group were moved in the 2 weeks before sampling	Dichotomous; baseline “no”
*cattle brought on (CBO)*	Whether or not cattle were moved on to the farm in the year before sampling	Dichotomous; baseline “no”
*breeding females brought on (BFBO)*	Whether or not breeding female cattle were moved onto the farm in the year before sampling.	Dichotomous; baseline “no”
*fatteners brought on (FBO)*	Whether or not fattening cattle had been moved onto the farm in the year before sampling.	Dichotomous; baseline “no”
*livestock on farm not owned by farmer*	Whether or not there were livestock of any species present on the day of sampling, which were not owned by the respondent (e.g. over-wintering sheep)	Dichotomous; baseline “no”
*bought other livestock*	Whether or not any livestock (not cattle) had been purchased in the year before sampling	Dichotomous; baseline “no”
*cattle elsewhere*	Whether or not employed farm workers caring for cattle on the sampled farm could also come into contact with cattle through work or family/friends elsewhere.	Dichotomous; NotApp answers categorised as “no”; baseline “no”
*non mains water*	Whether or not sampled cattle had access to non-mains water	Dichotomous; baseline “no”
*organic*	Whether or not routine farm practice was to spread cattle manure or slurry on grazing/silage ground	Dichotomous; NotApp answers categorised as “no”; baseline “no”
*wild geese*	Whether or not flocks of 100 or more geese had been seen on fields to which cattle have access	Dichotomous; baseline “no”
*gulls*	Whether or not flocks of 100 of more gulls had been seen on fields to which cattle have access	Dichotomous; baseline “no”
*ewes*	Whether or not there were breeding ewes present on the farm on sampling day	Dichotomous; baseline “no”
*survey*^b^	Whether or not the survey was conducted in Scotland or in England & Wales	Dichotomous; baseline “no”
*stx status*^c^	Indicating which Shiga toxins were present in faecal pat samples collected from a positive farm	Categorical, 3 levels: stx1&stx2_neg (baseline); stx1neg_stx2pos; stx1&stx2pos
*percent pos*^c^	The percentage of all faecal pat samples collected from a positive farm, which tested individually positive for *E. coli* O157	Continuous

The majority of PRFs on this list were tested for all three outcomes for interest

*NotApp* Not applicable, *PRF*, potential risk factor; *stx* shiga toxin

^a^ PRF only used for the analysis of Scotland data

^b^ PRF only used for the analysis of the combined data sets from both surveys

^c^ PRF only used for the analysis of whether or not a super-shedder sample was present in samples from a positive farm (Outcome 3)

#### Outcome 1 – all farms – farm classified as positive for *E. coli* O157

For the Scotland data, four variables with statistically significant effects were included in the final multivariable model (Table 2). In this model there was an increased odds ratio for a farm being classified as positive for *E. coli* O157 if breeding females were moved onto the farm in the past year (*BFBO*). There was also a slight increase in the odds ratio of a farm having *E. coli* O157 positive status for each additional animal in the 12–30 months age group (*cattle12–30 months*). There was a seasonal effect (*season*): if sampling was carried out in spring (March–May), the odds ratio was less than one, when compared to the baseline of sampling in autumn (September–November). The variable related to the introduction of livestock other than cattle onto the farm in the previous year (*bought other livestock*) was also found to be associated with reduced odds ratio for a farm being classified as positive.

**Table 2:** Final multivariable logistic model for Outcome 1^a^ for the Scotland data

Variable	Value	Coefficient	OR [95% CI]	*p*-value
Intercept		−1.04		0.040
*breeding females brought on (BFBO)*	No	Baseline	1.00	
	Yes	1.71	**5.52** [1.81–16.89]	**0.003**
*cattle 12–30 months*		0.009	**1.009** [1.001–1.02]	**0.028**
*bought other livestock*	No	Baseline	1.00	
	Yes	−1.45	**0.23** [0.07–0.75]	**0.015**
*season*	Autumn	Baseline	1.00	
	Winter	−0.53	0.59 [0.16–2.20]	0.433
	Spring	−2.33	**0.10** [0.02–0.56]	**0.009**
	Summer	−0.45	0.64 [0.18–2.30]	0.493

*OR* Odds ratio; *CI* Confidence Interval

Significant (*p* ≤ 0.05) OR and *p*-values are highlighted in bold text

McFadden’s pseudo *R*^2^ = 0.21

^a^ Farm classified as positive for *E. coli* O157

In comparison, the final model for the England & Wales data included one statistically significant variable, *total cattle*, which increased the odds ratio of a farm being classified as positive for *E. coli* O157 (Table 3).

**Table 3:** Final multivariable logistic model for Outcome 1^a^ for a. the England & Wales data; b. the combined data sets

		a. England & Wales			b. Scotland + England & Wales		
Variable	Value	Coefficient	OR [95% CI]	*p*-value	Coefficient	OR [95% CI]	*p*-value
Intercept		−2.03		< 0.0001	−1.71		< 0.0001
*total cattle*		0.005	**1.005 [1.002–1.009]**	**0.001**	0.003	**1.003 [1.001–1.004]**	**0.008**
*survey*	Scotland	N/A			Baseline	1.00	
	England & Wales				0.08	1.083 [0.587–1.999]	0.798

^a^ Farm classified as positive for *E. coli* O157

*OR* Odds ratio; *CI* Confidence Interval; *N/A* not applicable

Significant (*p* ≤ 0.05) OR and *p*-values are highlighted in bold text

Model a. McFadden’s pseudo R2 = 0.09; Model b. McFadden’s pseudo R2 = 0.03

When both data sets were combined, the final selected model was essentially a mirror of the final model for the England & Wales data alone, with the addition of *survey* as a non-significant variable that was essential to account for differences between the two surveys (Table 3).

#### Outcome 2 – positive farms only – the proportion of pats that tested individually positive for *E. coli* O157

Housing status of the sampled group (*housed)* was the sole statistically significant variable for both the Scotland data (Table 4) and England & Wales data (Table 5) for this outcome. A sampled group being housed at the time of sampling was associated with an increase in pat-level prevalence within the group. In the combined data sets, *survey* was also a statistically significant variable; farms in the England & Wales data had lower pat-level prevalence than farms in the Scotland data (Table 5).

**Table 4:** Final logistic model for Outcome 2^a^ for the Scotland data

Variable	Value	Coefficient	OR [95% CI]	*p*-value
Intercept		−1.78		0.004
*housed*	No	Baseline	1.00	
	Yes	2.21	**9.10** [2.65–31.25]	**0.002**

Significant (*p* ≤ 0.05) OR and *p*-values are highlighted in bold text

Unadjusted deviance explained by the model = 0.37

^a^ The proportion of pats on positive farms that tested individually positive for *E. coli* O157, *OR* Odds ratio; *CI* Confidence Interval

**Table 5:** Final multivariable logistic model for Outcome 2^a^ for a. the England & Wales data; b. the combined data sets

		a. England & Wales			b. Scotland + England & Wales		
Variable	Value	Coefficient	OR [95% CI]	*p*-value	Coefficient	OR [95% CI]	*p*-value
Intercept		−2.20		0.0003	−1.63		0.0002
*housed*	No	Baseline	1.00		Baseline	1.00	
	Yes	1.76	**5.81** [1.82–18.49]	**0.006**	2.02	**7.51** [3.25–17.37]	**< 0.0001**
*survey*	Scotland	N/A			Baseline	1.00	
	England & Wales				−0.80	**0.451** [0.24–0.86]	**0.018**

*OR* Odds ratio; *CI* Confidence Interval; *N/A* not applicable

Significant (*p* ≤ 0.05) OR and *p*-values are highlighted in bold text

Model a, Unadjusted deviance explained = 0.25

Model b, Unadjusted deviance explained = 0.35

^a^ The proportion of pats on positive farms that tested individually positive for *E. coli* O157

#### Outcome 3 – positive farms only – presence of at least one super-shedder pat

In the Scotland data, three variables had univariable associations with this outcome, but none were retained in any multivariable logistic model. In the England & Wales data, a final multivariable model of all individually significant factors could not be fitted due to the relatively small number of farms with super-shedder status. Several combinations of factors were not represented in the data; therefore, the variables *feed changed* and *housed* were not considered as candidate variables in the multivariable analysis, despite being associated with the outcome at a univariable level. In the final model *percent pos* was the only statistically significant variable (Table 6). In the analysis of the combined survey data, the only retained significant variable was *percent pos* (Table 6).

**Table 6:** Final multivariable logistic model for Outcome 3^a^ for a. the England & Wales data; b. the combined data sets

		a. England & Wales			b. Scotland + England & Wales		
Variable	Value	Coefficient	OR [95% CI]	*P*-value	Coefficient	OR [95% CI]	*P*-value
Intercept		−3.24		0.006	−2.246		0.002
*percent pos*		0.045	**1.046** [1.012–1.080]	**0.007**	**0.032**	**1.03** [1.01–1.05]	**0.002**
*survey*	Scotland	N/A			Baseline	1.00	
	England & Wales				−0.355	0.70 [0.19–2.58]	0.59

Significant (*p* ≤ 0.05) OR and *p*-values are highlighted in bold text

*OR* Odds ratio; *CI* Confidence Interval; *N/A* not applicable

Model a. McFadden’s pseudo R2 = 0.27; Model b. McFadden’s pseudo R2 = 0.18

^a^ Presence of at least one super-shedder sample on *E. coli* O157 positive farms

### Model comparison

When the final multivariable model for Outcome 1 (farm classified as positive for *E. coli* O157) from the Scotland data was fitted to the England & Wales data, none of the four variables that had been statistically significant in the Scotland model remained significant (Table 7). The variable *cattle 12–30 months* approached the threshold for statistical significance (*p* = 0.075). It was highly correlated (Additional file 4) with *total cattle* in both the England & Wales and Scotland data. In the England & Wales final multivariable model *total cattle* was a statistically significant variable for this outcome (Table 3).

**Table 7:** Final multivariable logistic model for Outcome 1^a^ for the Scotland data fitted to the England & Wales data

		Scotland			England & Wales		
Variable	Value	Coefficient	OR [95% CI]	*p*-value	Coefficient	OR [95% CI]	*p*-value
Intercept		−1.04		0.04	−2.088		< 0.0001
*Breeding females brought on (BFBO)*	No		Baseline			Baseline	
	Yes	1.71	**5.52** [1.81–16.89]	**0.003**	−0.204	0.815 [0.34–1.94]	0.643
*cattle 12–30 months*		0.009	**1.01** [1.001–1.02]	**0.028**	0.007	1.007 [0.10–1.02]	0.075
*bought other livestock*	No	Baseline	1.00		Baseline	1.00	
	Yes	−1.45	**0.23** [0.07–0.75]	**0.015**	0.613	1.846 [0.80–4.28]	0.153
*season*	Autumn	Baseline	1.00		Baseline	1.00	
	Winter	−0.53	0.59 [0.16–2.20]	0.433	0.517	1.68 [0.60–4.66]	0.322
	Spring	−2.33	**0.10** [0.02–0.56]	**0.009**	−0.046	0.96 [0.28–3.21]	0.941
	Summer	−0.45	0.64 [0.18–2.30]	0.493	0.546	1.73 [0.60–4.95]	0.310

^a^ Farm classified as positive for *E. coli* O157

*OR*, Odds ratio; *CI* Confidence Intervals

Significant (*p* ≤ 0.05) ORs and *p*-values are highlighted in bold text

## Discussion

The data collected from the BECS study, consisting of two cross-sectional prevalence surveys, offered a novel opportunity to investigate factors associated with *E. coli* O157 occurrence on equivalent farms in Scotland and in England & Wales, during the same time period. BECS was primarily designed to estimate prevalence within certain parameters and the sampled farms were demonstrated to be representative of the target population in both areas [20]. It was not designed as a study to investigate risk factors; hence the sample size may not have had the power to detect associations that exist between the potential risk factors and the various outcomes [21]. Nevertheless, some statistically significant associations were found. In some cases, these findings are consistent with the results of previous risk factor studies carried out within Great Britain; in other cases, the findings appear inconsistent. Differences in study design – e.g. case definitions, sampling protocols and target populations – are likely to contribute, at least in part, to the differences found between data from the BECS study and data from previous risk factor analyses; they could also be genuine differences or could be explained by chance (e.g. due to multiple testing).

Associations found in the Scotland data relating to on-farm occurrence of *E. coli* O157 (Outcome 1) are consistent with the results of other risk factor studies in Scotland. Farms that acquired breeding females [19] and those with greater numbers of cattle aged 12 to 30 months (*cattle 12–30 months*) were more likely to test positive for *E. coli* O157 [11]. Whilst the same specific effect of the number of cattle aged 12 to 30 months was significant neither in the multivariable analysis for the England & Wales data nor in the combined data sets, a significant positive association was found between herd size, when expressed as the total number of cattle present on the farm, and the presence of *E. coli* O157. These two variables (*total cattle* and *cattle 12–30 months)* were strongly correlated for Outcome 1 in the England & Wales data and the combined data sets. Thus, across both the individual and combined data sets, a measure of cattle numbers was associated with positive farm *E. coli* O157 status.

Previously, the number of cattle within a sampled group has been associated with increased within-group *E. coli* O157 prevalence in Scotland [11]. This finding was not supported by the results of the current analysis for Outcome 2 (the proportion of pats positive within positive groups). Sampling in the BECS survey was designed based on a previous large-scale cross-sectional survey in Scotland to have a mean 90% probability of detecting at least one positive pat if at least one animal in the sampled cattle group were shedding *E. coli* O157 at the time of sampling [22]. This protocol assumed a prevalence of 8% within positive groups, which was exceeded in both the IPRAVE and BECS studies [22]. Whilst the presence of more cattle within a positive group results in greater opportunity for more animals to be colonized with *E. coli* O157 and have the potential to shed the bacteria into their faecal pats, the intermittent nature of faecal shedding [23, 24] means that there is no guarantee that greater numbers of potentially colonized cattle will be reflected in the proportion of sampled pats that test positive for *E. coli* O157.

Although housed sample groups were no more likely to test positive for *E. coli* O157 than grazing sample groups, positive groups that were housed at the time of sampling had higher pat-level prevalence than positive grazing groups. This is consistent with previous research in Scotland [11]. It is biologically plausible that contact and thus transmission of *E. coli* O157 between animals is more likely when they are housed, leading to more animals shedding the bacteria at any one time point. Alternatively, the housing environment may improve conditions for bacterial survival and transmission due to faecal contamination, as has been described elsewhere [5, 25]. Although a cross-sectional study in England & Wales did not investigate whether the probability of positive *E. coli* O157 status among housed groups was increased compared to those at grass, it did demonstrate an association between poor condition of bedding material and group *E. coli* O157 status [26]. It is possible that the influence of housing in the current analysis could reflect a similar effect.

Principal drivers of when to house cattle, over and above the type of production system, relate to the quantity and quality (e.g. in terms of drainage, grazing cover) of land available to the producer, as well as geographical and topographical conditions, weather and climate in which the farm is located. Unless cattle numbers and/or land use change to a great degree, and always allowing for individual farm-related variation, it is reasonable to assume that the pattern of cattle housing is primarily dictated by fluctuating weather conditions. Weather conditions – certainly in a temperate zone such as the UK – can change substantially from year to year. Seasonality in the dynamics of *E. coli* O157 on cattle farms has been demonstrated in GB [11, 14], which is consistent with the findings for Outcome 1 for the Scotland data. Both *season* and *housed* were considered as candidates for inclusion in the multivariable models for Outcome 2, having shown univariable associations with this outcome across the individual and combined data sets. Ultimately, *housed* was the only statistically significant variable retained in the final model for Scotland and for England & Wales; it was retained alongside *survey* for the combined data sets. In the England & Wales data and the combined data there was a statistically significant association between *season* and *housed*, which was not the case for the Scotland data. One conclusion from these findings is that the particular effect of housing on the dynamics of *E. coli* O157 in cattle goes beyond a proxy seasonal effect and may indeed relate to the previous point regarding contamination of bedding or the cattle’s environment more generally. The apparent absence of any association between season and housing status in the Scotland data may reflect management factors that could not fully be explored in this analysis.

For the England & Wales data and the combined data the proportion of samples positive, i.e. within-group prevalence, was the only significant variable retained in the final model for the presence of a super-shedder within a positive group (Outcome 3). It is plausible that the more positive samples there are within a group, the greater the chance that one will be in the super-shedder category. Equally, this finding could support existing literature proposing that high-level shedding by one or more animals within a cattle group is associated with greater probability of low-level shedding by other members of that group [18, 19]. A model in which a small proportion of cattle within a group have a higher transmission rate for *E. coli* O157 has been proposed to best explain the distribution of *E. coli* O157 within cattle groups [7]. Whilst the results from the Scotland data did not appear consistent with this finding, this is likely to relate to sample size considerations and specifically to the limited number of positive farms with super-shedder status (*n* = 9 out of 26). There could also be some influence due to the particular subtypes of *E. coli* O157 in each location [27].

The current analysis demonstrated univariable associations (at *p* ≤ 0.20) between farms classed as ‘specialist finisher’ (compared to the farms classed as ‘dairy‘) and farm *E. coli* O157 status (Outcome 1) for both the Scotland data (positive association) and the England & Wales data (negative association), but the respective effects were not seen following multivariable analysis. This supports evidence from the literature of no association between management type and farm *E. coli* O157 status in GB [5, 11] but contradicts findings from studies carried out elsewhere [28, 29]. An imbalance in the data with regard to representation of different management types [20] may have prevented demonstration of management effects, or this could relate to sample size. This analysis did, however, demonstrate a statistically significant association between a measure of cattle numbers and positive farm *E. coli* O157 status, as well as a statistically significant association between a measure of cattle numbers and *management type* in Scotland and in England & Wales. It is possible that an underlying relationship between management type and the presence of *E. coli* O157 was present in these data and that herd size (*total cattle*) is acting as a proxy for other unknown and unquantified management factors within the sampled farms.

In this study, farms in Scotland on which livestock other than cattle had been bought in the year prior to sampling were less likely to test positive for *E. coli* O157 (Outcome 1) than farms where this had not occurred. The same effect was not detected in the data from England & Wales. In Scotland, *bought other livestock* was positively associated, at univariable level, with breeding female sheep being present on the farm (Additional file 4: Table S4) and was associated – though not at the level of *p* ≤ 0.05 – with cattle having access to water from a non-mains source (data not shown). This may indicate that *bought other livestock* could be acting as a proxy measure and that these farms are more likely to be extensive in nature. This can only be hypothesized as the questionnaire did not acquire this information directly. The Scottish cattle industry has a much greater extensive component than England, if not Wales, due to the proportion of Scottish land designated as “Less Favoured Area” [30]. If it were the case that these Scottish farms were more extensive, then the apparent protective effect of *bought other livestock* could relate not only to the possibility that extensive cattle herds may be more stable, with less mixing, lower stocking densities and other factors that might reduce the potential for introduction and within-herd transmission (maintenance) of *E.coli* O157, but also to the possibility that such cattle on such farms may be less exposed to stressors that can trigger them to shed the bacteria, once colonized.

Associations have been described elsewhere between the age of sampled animals and the probability of an individual or group testing positive for *E. coli* O157 [11, 15, 29]. In the Scotland data there was an increased pat-level prevalence within positive groups as the age of the oldest animal in the group increased, but this effect was only apparent at the univariable level and was not retained in multivariable analysis. Apart from this, no age effects were found in the current study. In positive farms in Scotland, *oldest in group* was significantly associated with *season*. There is potential for a complex interrelationship between housing status, age and season – amongst other factors – due to typical calving and management patterns for cattle in the UK that are intended for beef production. Two distinct calving periods are recognised in the UK: spring calving and late summer/autumn calving [31]. The season of birth will influence the age at which the calf is out at pasture and, therefore, the age at which it is housed, which means there is the potential for effects associated with housing and season to relate also to age. In the analyses described here an association between housing status and the ages of the oldest or youngest animals in the sample group was not found in either survey or in the combined data. Whether or not age is found to be a risk factor for the presence of *E. coli* O157 among a group of cattle or the proportion of samples collected from a group which test positive for *E. coli* O157 will also depend on how age is categorised in the analysis in question and on the sampling approach. The current study differed in these regards from other work where associations have been found between *E. coli* O157 status and age expressed as a categorical variable [14, 28] and where the target population has been older animals [29]. This, in addition to the lack of power in this study to be able to tease out complex multifactorial effects, could contribute to explaining why age-related PRFs were not retained in the final multivariable analyses, despite having been found to be a risk factor in previous research.

The study that provided the data for these analyses offered a unique opportunity to evaluate potential risk factors for *E. coli* O157 on cattle farms across Scotland and England & Wales through two contemporaneous surveys of similar design. Whilst, as a cross-sectional study, the design precludes any conclusions relating to causality being drawn, there are associations identified that can lead to the development of hypotheses. These hypotheses could then be further investigated as potential control or risk mitigation measures and may be important for understanding the dynamics of *E. coli* O157 on cattle farms. Despite a small sample size, it has been possible to demonstrate associations between certain demographic and management factors and the outcomes of interest – notably a) the total number of cattle on the farm and the presence of *E. coli* O157 on farms and b) the housing status of the sampled cattle and the level of *E. coli* O157 presence within positive groups. The suggestion that different factors may be more or less important depending on whether the question relates to the presence of *E. coli* O157 on a farm to begin with, or the extent to which it is found among samples from positive groups, is, in itself, a valuable finding. It may be the case that measures to protect public health by reducing the risk from cattle should be tailored according to whether it is desirable to prevent entry of *E. coli* O157 to a farm or deal with its presence on a farm that is already positive. It is also notable that farm-level risk factors differ between Scotland and England & Wales. This was supported by the outputs when the final model for the Scotland data was applied to the data from England & Wales. Whilst the herd-level prevalence of *E. coli* O157 did not differ significantly for Scotland and England & Wales [20], farms sampled in England & Wales were associated with a lower prevalence of *E. coli* O157 in positive groups. Reasons for this could relate to environmental or management effects as well as characteristics of the particular subtypes of *E. coli* O157 isolated, if there is regional variation in their prevalence within Great Britain. If such variation exists, it may be appropriate to issue region-specific advice on managing *E. coli* O157 risk among cattle groups, rather than assuming that a GB-wide protocol is the best approach. This is particularly the case where cattle producers already have a range of pathogens to consider in their herd health plan. Any recommended management approaches to mitigate the risk of *E. coli* O157 at farm level would compete for time and resources with a range of other endemic cattle diseases. Many of these have a greater production and welfare impact on the cattle themselves and would arguably take higher priority for producers. It is therefore crucial to ensure that the advice given is relevant to the farm under consideration.

The observation that farm-level risk factors for *E. coli* O157 presence in cattle intended for the food chain may differ between Scotland and England & Wales has further importance in relation to the Harmonised Epidemiological Indicators (HEI) for pathogenic verocytotoxin-producing *E. coli* (VTEC) for meat inspection, proposed by the European Food Safety Authority (EFSA) [32, 33]. It is proposed that information on the three farm HEIs – (HEI 1: practices which increase the risk of introducing pathogenic VTEC into the farm; HEI 2: on-farm practices and conditions; HEI 3: pathogenic VTEC status of the bovine animals to be slaughtered within one month) – would provide a pre-slaughter risk categorisation for pathogenic VTEC of incoming animals to the slaughterhouse. Awareness that there are likely to be differences in farm-level risk factors across different countries/regions highlights the need for further studies to identify country/region-specific risk factors that could be incorporated in HEIs, thus ensuring that risk categorisations for pathogenic VTEC are appropriate.

Additional investigations are underway: the isolates collected in these two surveys are being characterised at the molecular level to investigate and classify circulating cattle strains across Great Britain. This will allow comparisons to be made between the strains isolated in Scotland and in England & Wales. These isolates from the BECS study will also be compared with those collected during two previous Scottish cross-sectional surveys. Furthermore, planned comparisons of these cattle isolates with isolates from human clinical cases of *E. coli* O157 and the use of whole genome sequencing may identify important determinants of zoonotic potential.

## Conclusions

In this study, using data collected during two cross-sectional surveys of *E. coli* O157 in cattle intended for the food chain (in Scotland and in England & Wales), factors associated with detection of *E. coli* O157 in cattle groups were identified. The results suggest that certain risk factors may be important for the presence of *E. coli* O157 at farm level whilst other factors may have a role to play in the distribution – and thus the dynamics – of the organism within colonized cattle groups. Whilst factors associated with a positive farm status varied between Scotland and England & Wales, one consistent factor associated with positive farms was some measure of herd size. A cross-sectional survey cannot determine whether such associations are cause, or effect; however, their identification is valuable to contribute to risk mitigation efforts. Further studies will be needed in order to better understand *E. coli* O157 transmission within and between cattle herds. This will inform the discussion not only relating to how best to develop control strategies and interventions that can reduce the risk to human health from contact with cattle and their environment, but also whether or not this is the optimal point of intervention against *E. coli* O157.

## Methods

### Data

The laboratory results and questionnaire data used in these risk factor analyses were collected as part of the BECS cross-sectional surveys of the prevalence of *E. coli* O157 in cattle intended for the food chain. A detailed description of the aims, methodology and outcomes of the original study is available elsewhere [20]**.**

Briefly: faecal pat samples from the group of cattle closest to finishing were collected in two comparable surveys, from 110 farms in Scotland and 160 farms across England & Wales between September 2014 and November 2015. A questionnaire was administered at each farm visit to gather data on management, cattle demographics and specific information relating to the sampled cattle groups.

In Scotland, farms that had participated in both of two previous Scottish *E. coli* O157 prevalence studies [22] and that were still in business according to statutory registers were eligible for recruitment. In England & Wales, a sampling frame comparable to the original eligibility criteria for the Scottish studies - at least one non-dairy female or at least one male bovine aged over one year at the time of data retrieval - was obtained by random selection from all farms that met the eligibility criteria, according to statutory registers [20]. Initially all farms in the sampling frame were contacted by post to explain the background to the study, notify them that they might be contacted by phone and to provide them with the opportunity to opt out; for example, if they did not wish to participate, or the statutory register information was incorrect and they did not meet the eligibility criteria. Thereafter, a standardised telephone contact procedure was used to recruit farms, with farms randomly selected via a bespoke software programme, on an ongoing basis over the study period. There were four trained recruiters (two in each of Scotland and England & Wales). Farm visits to collect faecal pat samples and complete a questionnaire by face-to-face interview were conducted four field staff in Scotland and 10 field staff in England & Wales. Standardisation was achieved by an initial training day, followed by monthly teleconferences throughout the survey. The questionnaire (available from the corresponding author) was a shortened version of the one used in the Scottish survey between 2002 and 2004 [19, 34], amended for regional differences in terminology, then piloted and approved by the relevant bodies [20]. Completion of the questionnaire was electronically, through face-to-face interview at the farm visit. Data were captured from the electronic version into a database once the field visit was completed. All entered data were routinely checked centrally for anomalies and missing data and, if necessary, followed up with/by the field staff. Faecal pats were sampled in accordance with a protocol and sampling schedule used in the previous Scottish prevalence studies [19, 22, 34]. A universal container was filled almost to the top with small amounts of faeces taken from multiple locations on the surface of freshly voided faecal pats, found in the sample group’s environment. A pat was sampled only once. Immunomagnetic separation methods [35] were used to determine *E. coli* O157 status and a farm was defined as positive if at least one faecal pat sample was positive, as described in Henry et al. [20]. For further details of methods and results of analyses of recruitment and participation at each stage please see Henry et al. [20] and the final report of Food Standards Scotland project FS101055 [27].

### Outcomes of interest

There were three outcomes of interest:

Outcome 1 – all farms – whether or not the farm was classified as positive for *E. coli* O157 in the BECS study.

Outcome 2 – positive farms only – the proportion of pats that were classified as positive.

Outcome 3 – positive farms only – whether or not at least one pats was classed as a super-shedder, based on the count of *E. coli* O157 bacteria. A super-shedder pat was defined as 10^4^ CFU *E. coli* O157 g^− 1^ of faeces [8].

### Selection of potential risk factors

A list of potential risk factors (PRFs) was generated for each outcome from the laboratory and questionnaire data available. Inclusion in this list was based on published risk factors for *E. coli* O157 on cattle farms and/or because the PRF was considered to be biologically relevant.

### Statistical methods

All statistical analyses were performed using R version 3.4.2 [36]. Each outcome of interest was investigated individually for each of the Scotland data and the England & Wales data and then for the combined data sets. For analysis of the combined data sets, the variable *survey* was included a priori to account for possible differences between surveys.

All PRFs were individually screened to determine whether they were associated with the relevant outcome. PRFs that were associated with the outcome were retained for multivariable analysis. Multivariable analysis followed a stepwise forwards selection and backwards elimination procedure. The change in model deviance resulting from inclusion of a PRF was assessed for statistical significance. This was done using comparison of nested logistic models, for both PRF screening and multivariable analysis. The chi-square (χ^2^) test was used to select the preferred model at each stage. The threshold for statistical significance, and therefore retention, was *p* ≤ 0.20 in the PRF screening; in the multivariable analysis it was *p* ≤ 0.05.

### Individual survey data sets

#### PRF screening

The model structures for each of the three outcomes were as follows:

##### Outcome 1 – all farms – farm classified as positive for *E. coli* O157

Farm-level status (*farm class*) was defined as ‘1’ if the farm was positive in the BECS study, otherwise as ‘0’. Two logistic binomial models were compared, having the following structures:

logit (Pr*(*farm class*)) ~ intercept+PRF.

logit (Pr (*farm class*)) ~ intercept.

*Pr, Probability.

##### Outcome 2 – positive farms only – the proportion of pats that tested individually positive for *E. coli* O157

Visualisation of these data suggested that overdispersion may be present. This was confirmed by fitting a logistic model with only an intercept and comparing the total residual variance with the number of degrees of freedom, *p*-value < 10^− 100^ [37]. The quasi-binomial model was therefore selected as the most appropriate way of dealing with this feature [37, 38]. Two quasi-binomial models were compared, having the following structures:

logit (total positive pats/(total pats - total positive pats)) ~ intercept+PRF.

logit (total positive pats/(total pats - total positive pats)) ~ intercept.

##### Outcome 3 – positive farms only – presence of at least one super-shedder pat

The presence of a super-shedder (*supershedder*) pat gave a farm designation of ‘1’; if a positive farm contained no super-shedder pats it was designated ‘0’. Two logistic models were compared, having the following structures:

logit (Pr (s*upershedder*)) ~ intercept+PRF.

logit (Pr (*supershedder*)) ~ intercept.

#### Pairwise associations between retained variables

For each outcome of interest, all variables that were statistically significant in the PRF screening analysis were tested for association with all other statistically significant variables, using the complete and partial (*E. coli* O157 positive farms) data sets for Outcome 1 and Outcomes 2 and 3 respectively. Pearson’s product-moment correlation (PPMC), Fisher’s exact test (FET), linear regression (LR) and analysis of variance (AOV) were used, as appropriate.

#### Multivariable analyses

Logistic models for each outcome of interest were fitted using retained variables.

##### Forwards selection

A one-variable model containing the variable with the lowest *p*-value from PRF screening was compared to two-variable models by adding each of the other variables separately. The variable that resulted in the lowest p-value for change in model deviance was then included in the two-variable base model. This process was repeated until all variables retained after PRF screening had been tested and model fit could not be significantly improved by adding any further variable.

##### Backwards elimination

The model that had been constructed through the forwards selection procedure, containing *n* variables, was now compared to several models containing *n-1* variables, in which each of the variables that had been retained through forwards selection was removed one by one, with replacement. Backwards elimination stopped when removal of any remaining variables gave a statistically significant change in model fit. Odds ratios and their associated 95% Confidence Intervals were estimated in each final logistic model for factors statistically significantly associated with the relevant outcome.

##### Dealing with variable associations and interactions

Where variables that were retained in the final multivariable model were associated with others that were not retained in the final model, those alternative variables were substituted into the model and it was re-run. The choice of which variable should ultimately be included at the expense of the other was made based on model fit and on biological plausibility.

### Combined surveys (Scotland and England & Wales)

#### PRF screening

For each of the three outcomes, the process described above was repeated for the combined data sets.

#### Multivariable analyses

The approach for the stepwise procedure in the combined analyses followed that used for the individual data sets, except that the starting point for forwards selection was a two-variable model. This model included *survey* and the variable that had the lowest *p*-value for the association with the outcome, following PRF screening. The remaining analytical steps were the same as for the analyses of individual data sets.

### Model validation

The McFadden’s pseudo R^2^ was calculated to determine the proportion of the deviance that was explained by the model for Outcomes 1 and 3; for Outcome 2 the unadjusted deviance explained by the model was calculated.

### Model comparison

To further compare risk factors for Outcome 1 (farm classified as positive for *E. coli* O157) between Scotland and England & Wales, the final logistic model for the Scotland data was fitted to the England & Wales data.

## Supplementary information

**Additional file 1: Table S1.** Results of the PRF screening for Outcome 1

**Additional file 2: Table S2.** Reuslts of the PRF screening for Outcome 2

**Additional file 3: Table S3.** Results of the PRF screening for Outcome 3

**Additional file 4: Table S4.** Tests of association between pairs of significant PRFs in the PRF screening process for Outcomes 1, 2 and 3

## Data Availability

The datasets used and/or analysed during the current study have not been made publically available due to lack of permission from all of the study participants to do so. Where permissions have been granted data are available from the corresponding author on reasonable request.

## Abbreviations

AOV: Analysis of variance
BECS: British *E. coli* in Cattle Study
CFU g^− 1^: Colony-forming units per gram
CI: Confidence Interval
EFSA: European Food Safety Authority
FET: Fisher’s exact test
HEI: Harmonised Epidemiological Indicators
HUS: Haemolytic uraemic syndrome
LR: Linear regression
OR: Odds ratio
PPMC: Pearson’s product moment correlation
Pr: Probability
PRF: Potential risk factor
STEC: Shiga toxin-producing *Escherichia coli*
stx: Shiga toxin
VTEC: Verocytotoxin-producing *Escherichia coli*
χ2 test: Chi-square test
