# Supplementary material for: The British E. coli O157 in cattle study (BECS): factors associated with the occurrence of E. coli O157 from contemporaneous cross-sectional surveys
Source: BMC Vet Res. 2019 Dec 5;15:444. doi: 10.1186/s12917-019-2188-y (PMC6896709; doi:10.1186/s12917-019-2188-y)
Supplement: Supplementary file 1 — Additional file 1: Table S1. Results of the PRF screening for Outcome 1 [file 12917_2019_2188_MOESM1_ESM.docx]

**Table S1** Results of the PRF screening for Outcome 1*

|  |  | **Survey** | | | | | |
| --- | --- | --- | --- | --- | --- | --- | --- |
|  |  | **Scotland** | | **England & Wales** | | **England & Wales + Scotland**** | |
| **PRF** | **Value** | **OR**  **[95% CI]** | **p-value** | **OR**  **[95% CI]** | **p-value** | **OR**  **[95% CI]** | **p-value** |
| ***total cattle*** |  | 1.000  [0.997–1.003] | 0.98 | **1.01**  [1.00–1.01] | **0.001** | **1.003**  [1.00–1.00] | **0.01** |
| ***cattle 12 – 30 m*** |  | **1.01**  [1.00–1.01] | **0.09** | **1.01**  [1.00–1.01] | **0.05** | **1.006**  [1.00–1.01] | **0.01** |
| ***cattle less than 1 year*** |  | 1.00  [0.99–1.01] | 0.55 | **1.02**  [1.01–1.02] | **0.001** | **1.005**  [1.00–1.01] | **0.06** |
| ***group size*** |  | 0.99  [0.97–1.02] | 0.68 | **1.02**  [1.00–1.05] | **0.06** | **1.01**  [0.99–1.03 | **0.18** |
| ***management type*** | Dairy | 1.00 |  | 1.00 |  | 1.00 |  |
|  | Other | 1.44  [0.08–26.23] | 0.80 | 0.87  [0.19–2.42] | 0.86 | 1.05  [0.28–2.37] | 0.94 |
|  | Suckler beef | **4.57**  [0.56–37.36] | **0.16** | 0.68  [0.22–1.82] | 0.50 | 1.30  [0.50–2.25 | 0.59 |
|  | Specialist finisher | **8.12**  [0.80–82.73] | **0.08** | **0.14**  [0.0 –2.41] | **0.09** | 1.00  [0.28–2.27] | 0.99 |
| ***season*** | Autumn | 1.00 |  | Baseline |  | 1.00 |  |
|  | Winter | 0.48  [0.14–1.62] | 0.24 | 1.78  [0.66–2.77] | 0.26 | 1.03  [0.49–1.79] | 0.93 |
|  | Spring | **0.14**  [0.03–0.70] | **0.02** | 1.04  [0.32–2.23] | 0.94 | **0.44**  [0.17–1.37] | **0.09** |
|  | Summer | 0.75  [0.24–2.33] | 0.62 | 1.60  [0.58–2.62] | 0.37 | 1.15  [0.55–1.90] | 0.71 |
|  |  |  |  |  |  |  |  |
| ***housed*** | No | 1.00 |  | 1.00 |  | 1.00 |  |
|  | Yes | 0.66  [0.25–1.75] | 0.40 | **1.75**  [0.79–2.55] | **0.17** | 1.21  [0.65–1.83] | 0.55 |
| ***cattle brought on (CBO)*** | No | 1.00 |  | 1.00 |  | 1.00 |  |
|  | Yes | **4.00**  [0.87–18.37] | **0.07** | 1.03  [0.42–1.92] | 0.95 | 1.61  [0.76–2.36] | 0.22 |
| ***breeding females brought on (BFBO)*** | No | 1.00 |  | 1.00 |  | 1.00 |  |
|  | Yes | **3.09**  [1.25–7.67] | **0.01** | 0.98  [0.44–1.79] | 0.96 | **1.62**  [0.89–2.21] | **0.11** |
| ***livestock on farm not owned by farmer*** | No | 1.00 |  | 1.00 |  | 1.00 |  |
|  | Yes | 0.55  [0.15–2.08] | 0.38 | **0.15**  [0.02–2.19] | **0.07** | **0.33**  [0.11–0.96] | **0.04** |
| ***bought other livestock*** | No | 1.00 |  | 1.00 |  | 1.00 |  |
|  | Yes | **0.42**  [0.17–1.05] | **0.06** | 1.64  [0.75–2.43] | 0.22 | 0.92  [0.52–1.63] | 0.77 |
| ***cattle elsewhere*** | No | 1.00 |  | 1.00 |  | 1.00 |  |
|  | Yes | 1.56  [0.64–3.76] | 0.33 | **0.15**  [0.02–2.19] | **0.07** | **1.59**  [0.89–2.85] | **0.12** |
| ***organic*** | No | 1.00 |  | 1.00 |  | 1.00 |  |
|  | Yes | 0.53  [0.20–1.43] | 0.21 | **2.69**  [0.88 3.81] | **0.08** | 1.22  [0.60–2.48] | 0.58 |
| ***ewes*** | No | 1.00 |  | 1.00 |  | 1.00 |  |
|  | Yes | **0.42**  [0.17–1.04] | **0.06** | 1.62  [0.70–3.76] | 0.26 | 0.88  [0.49–1.57] | 0.66 |

* Farm classified as positive for *E. coli* O157

** OR estimates for PRFs using the combined data sets were calculated with the inclusion of the factor *survey* to account for differences at survey level

PRF, potential risk factor; OR, Odds Ratio; CI, Confidence Interval.

OR and p-values are highlighted when p ≤ 0.20

PRFs are shown if they were statistically significant (p ≤ 0.20) in at least one data set, and were retained for multivariable analysis. All remaining PRFs as listed in Table S1 were screened for this outcome, but were not statistically significantly associated with the outcome in either data set or in the combined data sets.
