# Supplementary material for: The British E. coli O157 in cattle study (BECS): factors associated with the occurrence of E. coli O157 from contemporaneous cross-sectional surveys
Source: BMC Vet Res. 2019 Dec 5;15:444. doi: 10.1186/s12917-019-2188-y (PMC6896709; doi:10.1186/s12917-019-2188-y)
Supplement: Supplementary file 2 — Additional file 2: Table S2. Reuslts of the PRF screening for Outcome 2 [file 12917_2019_2188_MOESM2_ESM.docx]

**Table S2** Results of the PRF screening for Outcome 2*

| **PRF** | **Value** | **Survey** | | | | | |
| --- | --- | --- | --- | --- | --- | --- | --- |
|  |  | **Scotland** | | **England & Wales** | | **England & Wales + Scotland**** | |
|  |  | **OR**  **[95% CI]** | **P-value** | **OR**  **[95% CI]** | **P-value** | **OR**  **[95% CI]** | **P-value** |
| ***total cattle*** |  | **1.003**  [1.001–1.01] | **0.11** | 1.000  [0.998–1.002] | 0.90 | 1.0009  [0.999–1.003] | 0.28 |
| ***cattle less than 1 year*** |  | **1.007**  [1.00–1.02] | **0.20** | 1.00  [0.99–1.01] | 0.79 | 1.004  [1.00–1.01] | 0.24 |
| ***oldest in group*** |  | **1.09**  [1.02–1.16] | **0.02** | 0.99  [0.95–1.02] | 0.46 | 1.01  [0.98–1.04] | 0.43 |
| ***management type*** | Dairy | 1.00 |  | 1.00 |  | 1.00 |  |
|  | Other | 0.11  [0–313.84] | 0.60 | **0.20**  [0.03–1.39] | **0.11** | **0.19**  [0.03–1.38] | **0.11** |
|  | Suckler beef | 0.84  [0.05–14.24] | 0.91 | 0.52  [0.18–1.48] | 0.23 | 0.56  [0.20–1.54] | 0.26 |
|  | Specialist finisher | 3.04  [0.13–73.32] | 0.50 | 1.03  [0.10–10.33] | 0.98 | 1.71  [0.37–7.87] | 0.49 |
| ***season*** | Autumn | 1.00 |  | 1.00 |  | 1.00 |  |
|  | Winter | **12.58**  [2.74–57.73] | **0.004** | **4.48**  [1.12–17.84] | **0.04** | **5.75**  [2.28–14.51] | **0.0005** |
|  | Spring | 0.83  [0.09–7.43] | 0.87 | **6.09**  [1.31–28.34] | **0.03** | **3.37**  [1.03–10.97] | **0.05** |
|  | Summer | 1.79  [0.48–6.68] | 0.39 | **3.68**  [0.80–16.92] | **0.10** | **2.46**  [0.91–6.64] | **0.08** |
|  |  |  |  |  |  |  |  |
| ***housed*** | No | 1.00 |  | 1.00 |  | 1.00 |  |
|  | Yes | **9.10**  [2.65–31.25] | **0.002** | **5.81**  [1.82–18.49] | **0.01** | 7.51  [3.25–17.37] | **<0.0001** |
| ***feed changed*** | No | 1.00 |  | 1.00 |  | 1.00 |  |
|  | Yes | **0.30**  [0.06–1.47] | **0.15** | 0.72  [0.25–2.07] | 0.54 | **0.50**  [0.21–1.23] | **0.14** |
| ***location changed*** | No | 1.00 |  | 1.00 |  | 1.00 |  |
|  | Yes | 0.61  [0.13–2.84] | 0.54 | **0.50**  [0.18–1.36] | **0.18** | **0.54**  [0.23–1.28] | **0.17** |
| ***cattle brought on (CBO)*** | No | 1.00 |  | 1.00 |  | 1.00 |  |
|  | Yes | **28.50**  [0.26–3127.91] | **0.18** | 1.47  [0.53–4.03] | 0.47 | **2.34**  [0.88–6.24] | **0.09** |
| ***bought other livestock*** | No | 1.00 |  | 1.00 |  | 1.00 |  |
|  | Yes | **2.78**  [0.90–8.55] | **0.09** | 0.71  [0.28–1.81] | 0.48 | 1.40  [0.67–2.90] | 0.37 |
| ***non mains water*** | No | 1.00 |  | 1.00 |  | 1.00 |  |
|  | Yes | **3.65**  [0.70–18.91] | **0.14** | 1.43  [0.28–7.29] | 0.67 | **2.56**  [0.81–8.16] | **0.12** |
| **gulls** | No | 1.00 |  | 1.00 |  | 1.00 |  |
|  | Yes | **0.34**  [0.10–1.15] | **0.09** | 1.00  [0.36–2.81] | 0.99 | **0.58**  [0.26–1.29] | **0.19** |

* The proportion of pats on positive farms that tested individually positive for *E. coli* O157

** OR estimates for PRFs using the combined data sets were calculated with the inclusion of the factor *survey* to account for differences at survey level

PRF, potential risk factor; OR, Odds Ratio; CI, Confidence Interval

OR and p-values are highlighted when p ≤ 0.20

PRFs are shown if they were statistically significant (p ≤ 0.20) in at least one data set, and were retained for multivariable analysis. All remaining PRFs as listed in Table S1 were screened for this outcome, but were not statistically significantly associated with the outcome in either data set or when the data sets were combined.
