# Supplementary material for: The British E. coli O157 in cattle study (BECS): factors associated with the occurrence of E. coli O157 from contemporaneous cross-sectional surveys
Source: BMC Vet Res. 2019 Dec 5;15:444. doi: 10.1186/s12917-019-2188-y (PMC6896709; doi:10.1186/s12917-019-2188-y)
Supplement: Supplementary file 3 — Additional file 3: Table S3. Results of the PRF screening for Outcome 3 [file 12917_2019_2188_MOESM3_ESM.docx]

**Table S3** Results of the PRF screening for Outcome 3*

| **PRF** | **Value** | **Survey** | | | | | | | |
| --- | --- | --- | --- | --- | --- | --- | --- | --- | --- |
|  |  | **Scotland** | | **England & Wales** | | | | **England & Wales + Scotland**** | |
|  |  | **OR**  **[95% CI]** | **p-value** | **OR**  **[95% CI]** | | **p-value** | | **OR**  **[95% CI]** | **p-value** |
| ***group size*** |  | **1.06**  [0.99–1.14] | **0.08** | 0.975  [0.89–1.07] | | 0.58 | | 1.01  [0.98–1.04] | 0.55 |
| ***percent pos*** |  | **1.02**  [0.996–1.05] | **0.10** | **1.05**  [1.01–1.08] | | **0.007** | | 1.03  [1.01–1.05] | **0.002** |
| ***breeding females brought on (BFBO)*** | No | 1.00 |  | 1.00 | |  | | 1.00 |  |
|  | Yes | **0.27**  [0.05–1.50] | **0.14** | 1.78  [0.32–9.85] | | 0.51 | | 0.65  [0.19–2.24] | 0.50 |
| ***feed changed*** | No | 1.00 |  | 1.00 | |  | | 1.00 |  |
|  | Yes | 0.141  0.04–4.31 | 0.46 | OR*** |  | | | **0.18**  0.02–1.54 | **0.12** |
| ***housed*** | No | 1.00 |  | 1.00 | |  | | 1.00 |  |
|  | Yes | 0.84  0.15–4.72 | 0.84 | OR^***^ |  | | | **2.50**  0.61–10.25 | **0.20** |
| ***fatteners brought on (FBO)*** | No | 1.00 |  | 1.00 | | |  | 1.00 |  |
|  | Yes | 0.56  [0.11–3.02] | 0.50 | **4.25**  [0.69–26.13] | | | **0.12** | 1.48  [0.46–4.74] | 0.51 |

* Presence of at least one super-shedder sample on *E. coli* O157 positive farms

** OR estimates for PRFs using the combined data sets were calculated with the inclusion of the factor *survey* to account for differences at survey level

*** OR not defined because there are results with cells with ‘0’ values

PRF, potential risk factor; OR, Odds Ratio; CI, Confidence Interval.

OR and p-values are highlighted when p ≤ 0.20

PRFs are shown if they were statistically significant (p ≤ 0.20) in at least one data set, and were retained for multivariable analysis. All remaining PRFs as listed in Table S1, (Supplementary Information) were screened for this outcome, but were not statistically significantly associated with the outcome in either data set or in the combined data sets.
