# Supplementary material for: The British E. coli O157 in cattle study (BECS): factors associated with the occurrence of E. coli O157 from contemporaneous cross-sectional surveys
Source: BMC Vet Res. 2019 Dec 5;15:444. doi: 10.1186/s12917-019-2188-y (PMC6896709; doi:10.1186/s12917-019-2188-y)
Supplement: Supplementary file 4 — Additional file 4: Table S4. Tests of association between pairs of significant PRFs in the PRF screening process for Outcomes 1, 2 and 3 [file 12917_2019_2188_MOESM4_ESM.docx]

**Table S4** Tests of association between pairs of significant* PRFs in the PRF screening process for Outcomes 1**, 2*** and 3****

| **Outcome** | **Survey** | **PRF1** | **PRF2** | **Test of association** | **p-value** |
| --- | --- | --- | --- | --- | --- |
| 1 | Scotland | *cattle brought on* | *breeding females brought on* | FET | <0.0001 |
| 1 | Scotland | *bought other livestock* | *ewes* | FET | <0.0001 |
| 1 | Scotland | *cattle brought on* | *management type* | FET | 0.02 |
| 1 | Scotland | *management type* | *cattle 12-30 months* | AOV | 0.025 |
| 1 | Scotland | *cattle brought on* | *cattle 12-30 months* | LR | 0.009 |
| 1 | E & W | *total cattle* | *cattle 12-30 months* | PPMC | <0.0001 |
| 1 | E & W | *total cattle* | *group size* | PPMC | <0.0001 |
| 1 | E & W | *total cattle* | *cattle less than 1 year* | PPMC | <0.0001 |
|  | E & W | *total cattle* | *management type* | AOV | <0.0001 |
|  | E & W | *cattle 12-30 months* | *management type* | AOV | 0.01 |
|  | E & W | *cattle less than 1 year* | *management type* | AOV | 0.0002 |
| 1 | E & W | *cattle 12-30 months* | *group size* | PPMC | <0.0001 |
| 1 | E & W | *cattle 12-30 months* | *cattle less than 1 year* | PPMC | <0.0001 |
| 1 | E & W | *group size* | *cattle less than 1 year* | PPMC | <0.0001 |
| 1 | E & W | *housed* | *total cattle* | LR | <0.0001 |
| 1 | E & W | *housed* | *cattle 12-30 months* | LR | 0.007 |
| 1 | E & W | *housed* | *cattle less than 1 year* | LR | <0.0001 |
| 1 | E & W | *organic* | *non mains water* | FET | 0.046 |
| 1 | E & W | *organic* | *livestock on farm not owned by farmer* | FET | <0.0001 |
| 1 | E & W and Scotland | *cattle 12-30 months* | *cattle less than 1 year* | PPMC | <0.0001 |
| 1 | E & W and Scotland | *cattle 12-30 months* | *group size* | PPMC | <0.0001 |
| 1 | E & W and Scotland | *cattle 12-30 months* | *cattle brought on* | LR | 0.004 |
| 1 | E & W and Scotland | *cattle 12-30 months* | *total cattle* | PPMC | <0.0001 |
| 1 | E & W and Scotland | *cattle less than 1 year* | *group size* | PPMC | <0.0001 |
| 1 | E & W and Scotland | *cattle less than 1 year* | *breeding females brought on* | LR | 0.03 |
| 1 | E & W and Scotland | *breeding females brought on* | *cattle brought on* | FET | <0.0001 |
| 1 | E & W and Scotland | *total cattle* | *cattle less than 1 year* | PPMC | <0.0001 |
| 1 | E & W and Scotland | *total cattle* | *group size* | PPMC | <0.0001 |
| 1 | E & W and Scotland | *total cattle* | *cattle brought on* | LR | 0.004 |
| 1 | E & W and Scotland | *total cattle* | *breeding females brought on* | LR | 0.005 |
| 2 | Scotland | *total cattle* | *oldest in group* | PPMC | 0.007 |
| 2 | Scotland | *total cattle* | *cattle less than 1 year* | PPMC | <0.0001 |
| 2 | Scotland | *oldest in group* | *season* | AOV | 0.012 |
| 2 | E & W | *season* | *housed* | FET | 0.005 |
| 2 | E & W | *season* | *location changed* | FET | 0.04 |
| 2 | E & W and Scotland | *season* | *non mains water* | FET | 0.009 |
| 2 | E & W and Scotland | *cattle brought on* | *non mains water* | FET | 0.05 |
| 2 | E & W and Scotland | *cattle brought on* | *feed changed* | FET | 0.03 |
| 2 | E & W and Scotland | *season* | *housed* | FET | 0.006 |
| 3 | E & W | *percent pos* | *housed* | LR | 0.002 |
| 3 | E & W | *oldest in group* | *feed changed* | FET | 0.002 |
| 3 | E & W and Scotland | *percent pos* | *management type* | LR | 0.008 |
| 3 | E & W and Scotland | *percent pos* | *housed* | LR | <0.0001 |

* at p≤ 0.20

** Farm classified as positive for *E. coli* O157

^***^ The proportion of pats on positive farms that tested individually positive for *E. coli* O157

^****^ Presence of at least one super-shedder sample on *E.  coli* O157 positive farms

Only tests of association that were significant at p≤ 0.05 are shown

PRF = potential risk factor; FET = Fisher’s exact test; LR = Linear regression;

PPMC = Pearson’s Product Moment Correlation; AOV = Analysis of variance

E & W = England and Wales
